# *In silico* Optimization of Left Atrial Appendage Occluder Implantation Using Interactive and Modeling Tools

**DOI:** 10.3389/fphys.2019.00237

**Published:** 2019-03-22

**Authors:** Ainhoa M. Aguado, Andy L. Olivares, Carlos Yagüe, Etelvino Silva, Marta Nuñez-García, Álvaro Fernandez-Quilez, Jordi Mill, Ibai Genua, Dabit Arzamendi, Tom De Potter, Xavier Freixa, Oscar Camara

**Affiliations:** ^1^PhySense, Department of Information and Communication Technologies, Universitat Pompeu Fabra, Barcelona, Spain; ^2^Division of Interventional Cardiology, Hospital de la Santa Creu i Sant Pau, Universitat Autònoma de Barcelona, Barcelona, Spain; ^3^Arrhythmia Unit, Department of Cardiology, Cardiovascular Center, Aalst, Belgium; ^4^Department of Cardiology, Hospital Clínic de Barcelona, Universitat de Barcelona, Barcelona, Spain

**Keywords:** left atrial appendage occlusion, Computational Fluid Dynamics, *in silico* optimization of therapies, web-based implantation platform, atrial fibrillation

## Abstract

According to clinical studies, around one third of patients with atrial fibrillation (AF) will suffer a stroke during their lifetime. Between 70 and 90% of these strokes are caused by thrombus formed in the left atrial appendage. In patients with contraindications to oral anticoagulants, a left atrial appendage occluder (LAAO) is often implanted to prevent blood flow entering in the LAA. A limited range of LAAO devices is available, with different designs and sizes. Together with the heterogeneity of LAA morphology, these factors make LAAO success dependent on clinician's experience. A sub-optimal LAAO implantation can generate thrombi outside the device, eventually leading to stroke if not treated. The aim of this study was to develop clinician-friendly tools based on biophysical models to optimize LAAO device therapies. A web-based 3D interactive virtual implantation platform, so-called VIDAA, was created to select the most appropriate LAAO configurations (type of device, size, landing zone) for a given patient-specific LAA morphology. An initial LAAO configuration is proposed in VIDAA, automatically computed from LAA shape features (centreline, diameters). The most promising LAAO settings and LAA geometries were exported from VIDAA to build volumetric meshes and run Computational Fluid Dynamics (CFD) simulations to assess blood flow patterns after implantation. Risk of thrombus formation was estimated from the simulated hemodynamics with an index combining information from blood flow velocity and complexity. The combination of the VIDAA platform with *in silico* indices allowed to identify the LAAO configurations associated to a lower risk of thrombus formation; device positioning was key to the creation of regions with turbulent flows after implantation. Our results demonstrate the potential for optimizing LAAO therapy settings during pre-implant planning based on modeling tools and contribute to reduce the risk of thrombus formation after treatment.

## 1. Introduction

Atrial Fibrillation (AF) is the most common cardiac arrhythmia diagnosed in clinical practice, affecting around 0.4–1% of the general population (Singh and Holmes, 2010). The atria beat irregularly during AF, which leads to ineffective blood flow pumping to the ventricles. The consequences of AF have been clearly established in multiple studies, being one of them the increased risk of stroke; one out of three ischemic strokes is related to AF (Boston Scientific, 2018). Between 70 and 90% of AF-related strokes originate from thrombus formed in a cavity of the left atrium, the left atrial appendage (LAA) (Wang et al., 2010; Lee et al., 2015). The LAA is a contractile reservoir and a decompression chamber, depending on the cardiac cycle phase (Fatkin et al., 1994; Al-Saady et al., 1999). It acts as a suction during ventricular systole and during diastole as a conduit. In addition, the LAA is characterized by its high morphological variability, in parameters such as size, height, smoothness or number of lobes. The different LAA morphologies are often classified into four categories: chicken wing, cauliflower, windsock and cactus (Di Biase et al., 2012). Left atrial motion irregularity due to AF causes a deceleration of blood flow and stasis, increasing the risk of thrombus formation in the LAA (Markl et al., 2016). The thrombus formed can then travel through the circulatory system to the brain, causing a cardioembolic stroke.

**Figure 1:**
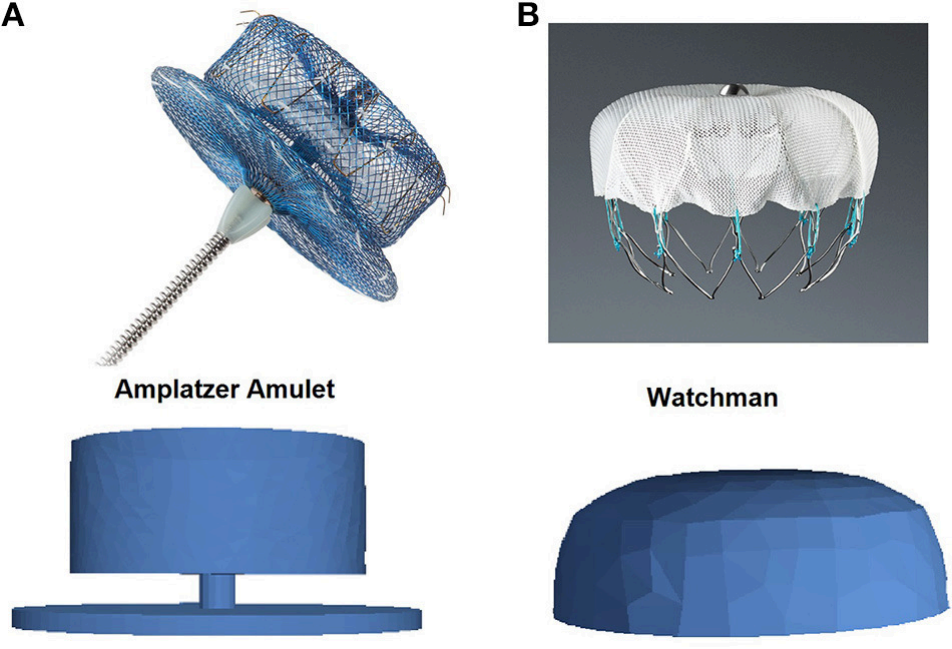
Pictures (top) and Computational-Aided Design models (bottom) of left atrial appendage occlusion devices. **(A)** Amplatzer Amulet (Abbott, 2018); **(B)** Watchman (Boston Scientific, 2018).

Oral anticoagulation (OAC) therapy is usually prescribed on those AF patients older than 65 years old (Morillo et al., 2017) to reduce the risk of thrombus formation. When OAC therapy is not appropriate, due to high risk of bleeding or because of patient's limitations, a physical LAA closure is performed with the implantation of a left atrial appendage occlusion (LAAO) device or surgery-based clipping (Le et al., 2014). LAAO devices are implanted percutaneously by a femoral vein approach under fluoroscopic and echocardiographic guidance. The most used LAAO devices available in the market (see Figure 1) are the Watchman (Boston Scientific, 2018) and the Amplatzer Amulet (Abbott, 2018), even if new promising devices such as the Lambre (Park et al., 2018) and the Wavecrest (Saw and Lempereur, 2014) are being proposed every year. In a LAAO procedure, the large intra-subject anatomical variation of the LAA influences the individual implantation. Therefore, biomedical images are acquired to have good anatomical knowledge of the cavity, before and during the intervention. In most clinical centers X-ray and transesophageal echocardiography (TEE) images are used to characterize the LAA morphology during the intervention to support decisions on device implantation. Other imaging modalities such as Computed Tomography (CT) (Chow et al., 2017) and 3D Rotational Angiography (3DRA) (De Potter et al., 2018) are also being explored in advanced hospitals to have higher-resolution structural information before and during the intervention, respectively. The ostium (interface betwen LA and LAA) dimensions and height/depth of the LAA cavity (Morillo et al., 2017) are critical LAA shape parameters to individualize the size of the implanted device and the landing zone (location where the device will be released). Appropriate sizing of the device, achieving a complete clinical closure, allows the procedure to be finished earlier since a second device is not required. A minimal leakage of jet blood flow entering into the LAA after device implantation is the standard criteria to define a successful closure (López-Mínguez et al., 2014). Unfortunately, LAA shape parameters are usually estimated from medical images with manual tools, being difficult to standardize criteria to objectively define them. Moreover, values of these parameters coming from different imaging modalities differ substantially due to their respective spatial resolution and limitations (López-Mínguez et al., 2014).

Beyond their morphology, studying LA and LAA hemodynamics is also important before and after LAAO treatment. The Virchow's triad defines blood hypercoagulability, hemodynamics changes and endothelial wall injury dysfunction as the three main players in thrombus formation (Chung and Lip, 2003). Left atrial appendage blood flow velocities below some threshold (<0.2 m/s) have been associated to the presence of thrombus in the LAA (Beigel et al., 2014). Unfortunately, most clinical studies (Beigel et al., 2014) involving the LAA, usually based on echocardiographic images, only report a single blood flow velocity value in one point in space (LAA ostium) and in time (end diastole), which constitutes an over-simplification of the complex hemodynamics in the LA and LAA. Detailed hemodynamics characterization is becoming possible these days with advanced 4D-flow magnetic resonance imaging technique, but there are few studies in the LA (Casas et al., 2017; Cibis et al., 2017a,b) and only one in the LAA (Markl et al., 2016). Some researchers (Zhang and Gay, 2008; Koizumi et al., 2015; Vedula et al., 2015; Otani et al., 2016; Olivares et al., 2017; Bosi et al., 2018; García-Isla et al., 2018) have developed biophysical modeling pipelines for a more detailed *in silico* analysis of LAA blood flow patterns with Computational Fluid Dynamics (CFD) simulations. However, none of these works studied the effect of implanting a LAAO device with different settings on atrial hemodynamics. In addition, as in the majority of biomedical applications, these biophysical models remain an engineering tool far from being used to support clinicians on their decisions.

Even if LAAO devices are generally becoming an accepted option when OAC therapy fails, it still exists controversy concerning the safety and the real clinical benefit of LAAO-based therapy. Although it seems to be effective, short- and long-term consequences are not fully known (Mandrola et al., 2018). As an example, a recent clinical trial (Fauchier et al., 2018) reported a high probability of getting a device-related thrombus (around 7%). Moreover, LAAO is recognized as a technically demanding procedure, requiring rigorous training and skills in order to reduce complications (Tzikas et al., 2016). However, there is a lack of advanced computational tools to provide a complete, objective and patient-specific characterization of LAA morphology and function to assist clinicians on the challenging LAAO device selection and planning steps. The aim of this work was to develop a clinician-friendly visualization tool to be combined with biophysical modeling for LAAO therapy optimization. A web-based 3D interactive virtual implantation platform, so-called VIDAA (Virtual Implantation and Device selection in left Atrial Appendages), was designed and implemented to jointly visualize the LA anatomy of an individual patient and different possible LAAO configurations. The VIDAA platform initially proposes LAAO setting parameters estimated after a morphological analysis of the LAA under study. The clinician later can interactively vary device parameters in VIDAA and export outside the platform the most promising configurations. Computational Fluid Dynamics (CFD) simulations were then run with a commercial solver for several LAAO configurations extracted from VIDAA to estimate their corresponding risk of thrombus formation after implantation. As a first proof of concept, the VIDAA platform was tested on retrospective data of four non-valvular AF patients that underwent a LAA closure.

## 2. Materials and Methods

**Figure 2:**
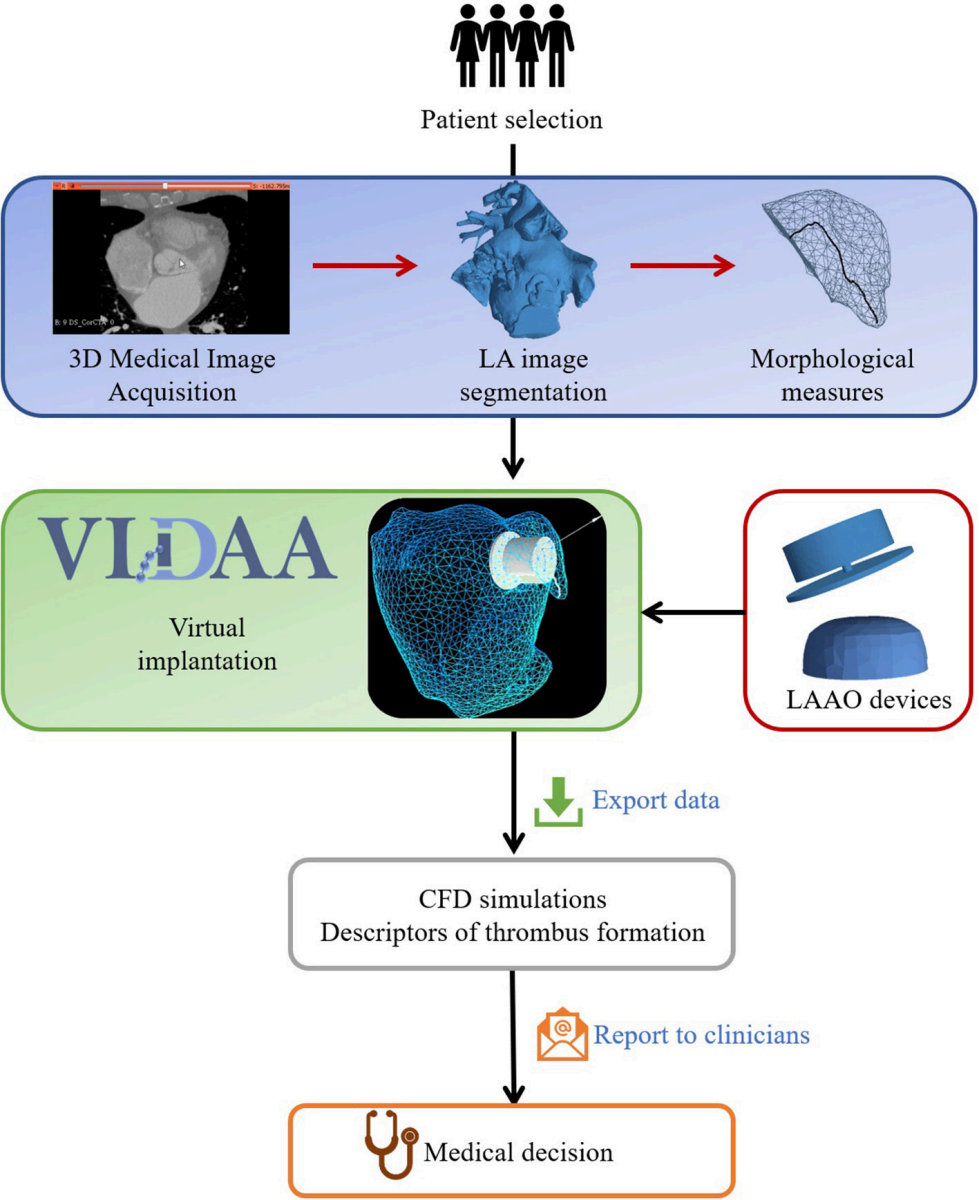
Computational workflow to recommend optimal left atrial appendage occlusion (LAAO) settings for a selected patient. The first stage produces LAA morphological measures from LA segmentations obtained on three-dimensional medical images. Computational 3D renderings of LA/LAA geometries are then jointly visualized with morphological measures and Computer-Aided Design models of LAAO devices in the VIDAA platform. The VIDAA platform allows an interactive and individualized virtual implantation of LAAO devices, generating multiple configurations that can be exported for further processing. Computational-Fluid Dynamics (CFD) simulations run on the exported LAAO configurations provide hemodynamics descriptors to estimate the risk of thrombus formation for each case, which are finally reported to clinicians to support their decisions.

The computational workflow designed in this work (see Figure 2) is a planning tool that, together with computational modeling, provides enhanced individualized information to the clinician before the intervention. In all analyzed cases, surface meshes of the LA were reconstructed from binary masks obtained segmenting high-resolution 3D medical images (CT and 3DRA). In the next step, morphological indices characterizing patient-specific LAA shapes were automatically calculated. Based on these LAA shape-based indices, a range of optimal LAAO device parameters (size, position) were estimated. The optimal configuration for the two most popular devices (e.g., Watchman and Amplatzer Amulet) could then interactively be explored together with a 3D rendering of the LA in the web-based VIDAA platform. The clinician could manually modify several LAAO parameters to better understand the case under study and plan the intervention. Once a given LAAO configuration was considered appropriate by the clinician, the VIDAA platform exported all data (e.g., meshes and their spatial relationship) that will define the geometrical domain where CFD simulations were run outside VIDAA with a commercial software. Simulation results were post-processed to derive *in silico* hemodynamics indices estimating the risk of thrombus formation for a given LAA and device setting. The VIDAA platform in combination with CFD simulations then allowed the clinician to decide, prior to the intervention, which device configuration was safer, in terms of thrombus formation.

### 2.1. Patient-Specific Imaging Data and Processing

Three-dimensional medical images were available for the four AF patients analyzed in this study: 3DRA images of three patients from OLV Hospital (Aalst, Belgium); and a CT image of one patient from Hospital Clínic de Barcelona (Spain). The OLV Hospital and Hospital Clínic de Barcelona ethical committees approved this study and written informed consent was obtained from every participant before the procedure. Watchman devices were implanted to patients at OLV Hospital while an Amplatzer Amulet was the choice at Hospital Clínic de Barcelona (see Table 1). All patients had persistent non-valvular atrial fibrillation with high risk of thrombus formation (e.g., CHAD2VASC > 2).

**Table 1:** Patient and device information.

**Patient**	**Medical image**	**CHAD2VASC**	**Device type**	**Device size (mm)**
1	3DRA	3	Watchman	27
2	3DRA	3	Watchman	30
3	3DRA	6	Watchman	27
4	CT	5	Amplatzer Amulet	22

*3DRA, 3D Rotational Angiography; CT, Computed Tomography; CHAD2VASC, clinical index characterizing the risk of thrombus formation*.

An Innova™ monoplane fluoroscopy system (GE Healthcare, LLC, Waukesha, WI) was used at OLV Hospital to acquire the 3DRA images, with 0.23 or 0.45 mm pixel size for 512 or 256 isotropic voxels, respectively. Before and during the injection, the right ventricle was burst paced at a cycle length of 200 ms during end-expiratory apnea to interrupt the blood flow through the LA. All datasets were segmented by an experienced operator on the Advantage Workstation (GE Healthcare) using the Innova 3D software to reconstruct the LA, which is based on threshold and region-growing semi-automatic segmentation techniques. The CT image at Hospital Clínic de Barcelona was acquired using a Siemens™ machine, synchronized with ECG and supplying 80 ml of contrast, having a volumetric pixel size of 0.33 × 0.33 × 0.40 mm for an in-plane matrix of 512 pixels and 430 slices. The ITK-Snap^1^ software (Yushkevich et al., 2006) was used to semi-automatically segment the LA in the CT image (also with region growing and threshold-based techniques, plus manual corrections). The LA binary masks from segmenting 3DRA and CT images were the basis to construct surface meshes with the Marching Cubes method for subsequent morphological analysis and simulations. A 2D plane was manually selected by the operator to define the ostium and the LAA in the LA 3D surface mesh.

#### 2.1.1. Left Atrial Appendage Morphological Parameters and Optimal Device Configuration

**Figure 3:**
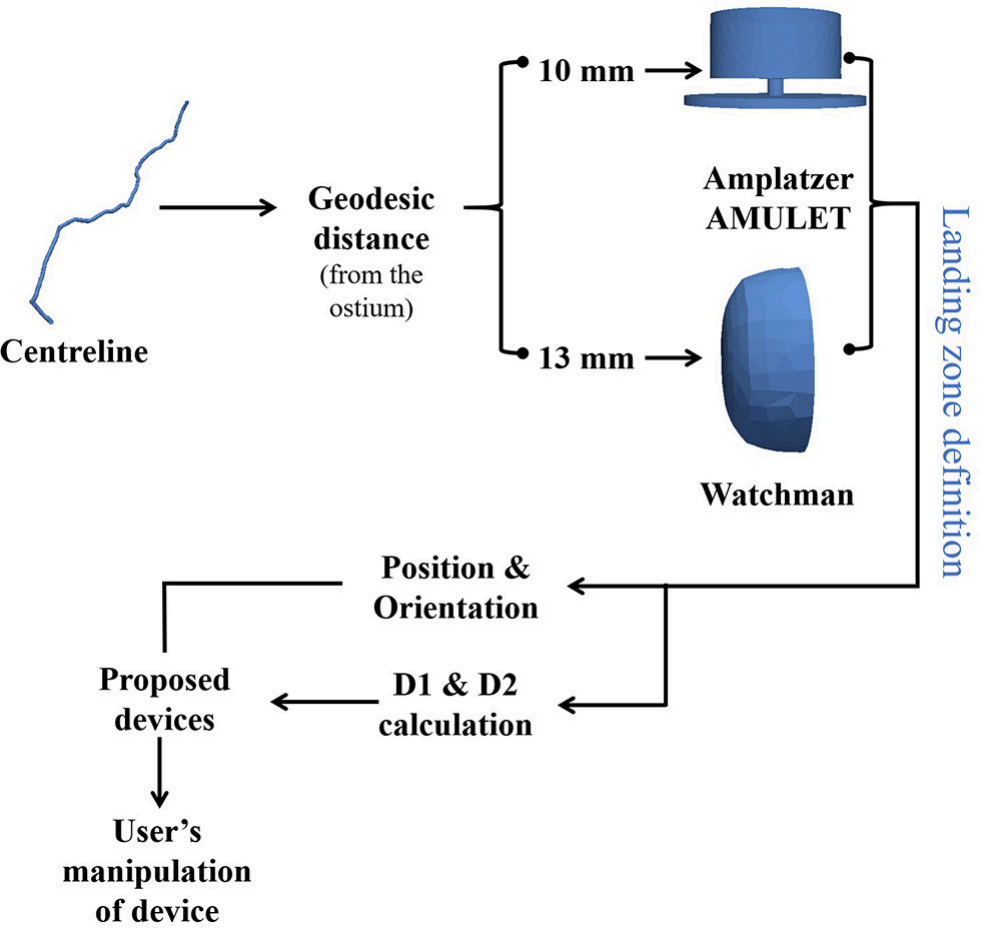
Computational pipeline to obtain optimal left atrial appendage occlusion (LAAO) device configuration. Device positioning (e.g., landing zone) is initially estimated at a certain geodesic distance from the ostium following the LAA centreline (10 and 13 mm for the Amplatzer Amulet and Watchman, respectively). Device orientation is set perpendicular to the LAA centreline. LAAO size is based on the maximum diameters (D1 & D2) of a transversal 2D plane perpendicular to the centreline placed in the optimal positioning. The proposed LAAO devices are initially shown in the VIDAA platform with these settings (VIDAA-init), where the clinician can interactively manipulate all their parameters for a better fitting to the studied LAA morphology (VIDAA-end).

Two types of morphological parameters were automatically estimated from LAA surface meshes: ostium- and centreline-based parameters. The ostium area, perimeter and principal axes were computed directly from surface mesh elements labeled as ostium after the segmentation. The LAA centreline (see and example in Figure 3) was obtained following a marching algorithm (Genua et al., 2019), where its starting point (the furthest point of the LAA) and the centreline direction to the ostium were computed applying a heat transfer propagation simulation to the LAA volumetric mesh. Once the centreline was estimated, several transversal 2D planes, perpendicular to its main direction, were selected along its length to compute their maximal (D1) and minimal (D2) diameters.

The centreline-based parameters were used to automatically estimate the optimal LAAO configuration for the analyzed anatomy, as illustrated in Figure 3. Following criteria defined by our experienced clinicians, device (center) positioning was set at a geodesic distance along the centreline of 10 and 13 mm from the ostium for the Amplatzer Amulet and the Watchman, respectively. The diameters (D1, D2) of the transversal 2D plane centered at the optimal device positioning were then computed. The initial orientation of the LAAO device was set perpendicular to the centreline. Based on empirical algorithms from clinicians and official device instructions, the most appropriate LAAO device sizes (three for the Amplatzer Amulet and two for the Watchman) were initially selected. For instance, optimal Amplatzer Amulet device sizes were established following the current clinical protocol at Hospital Clínic de Barcelona: 2–5 or 3–6 mm are usually added to the average mean diameter (between D1 and D2) if imaging data comes from CT or TEE, respectively. If the obtained size is smaller than D1, additional 2–5 mm are added. Finally, only devices ensuring at least a 10% of compression of original size after implantation were selected. Fulfilling the compression-based criteria increments the likelihood of getting the LAAO device attached to the wall for a successful closure, without creating too high stresses on the LAA. The optimal sizes for the Watchman device (see Table A.1 in Appendix 1) were selected based on D1, following official device instructions.

### 2.2. VIDAA: Virtual Implantation and Device Selection in Left Atrial Appendages

VIDAA is a web-based 3D interactive virtual implantation platform that allows clinicians to select the most appropriate LAAO configurations for a given patient. The main features of the VIDAA platform include: the joint 3D visualization of the LA/LAA surface meshes and the device models; different modes of mesh visualization and editing tools such as wireframe views or clipping, among others; transform controllers for user manipulation of the device such as translation, rotation and scaling; visualization of LAA morphological parameters such as the centreline, 2D transveral planes of the centreline and its diameters (D1, D2); visualization of the optimal LAA settings according to VIDAA such as device size and landing zone; manual modification of all LAAO settings; and export of a LAAO configuration for subsequent *in silico* hemodynamics study. Figure 2 illustrates the different steps in the VIDAA workflow. A demonstration video of a proof-of-concept prototype of VIDAA is also available as Supplementary Material (Appendix 2).

Being a web-based platform, VIDAA is not linked to any software installation, operative system or browser, which facilitates its usage in a clinical environment. VIDAA was built using HTML5 programming language (JavaScript, scss), using different JavaScript libraries such as THREE.js and React.js. The first library, THREE.js, was used to visualize and interact with 3D objects (e.g., LA mesh) while React.js was used to separate into components the different modalities of the platform.

### 2.3. Hemodynamics Simulations

#### 2.3.1. Mesh Processing Pipeline

**Figure 4:**
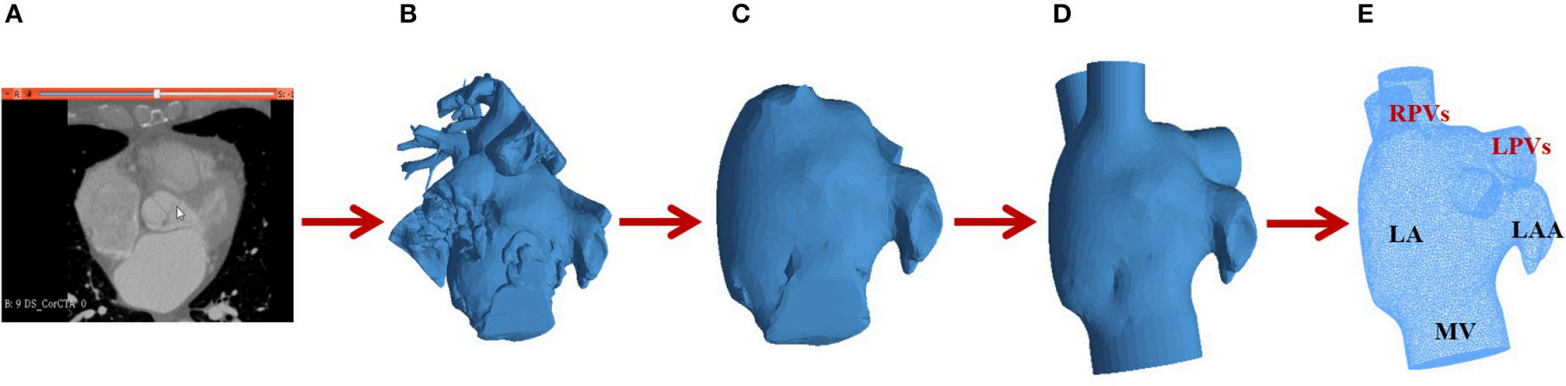
Mesh processing pipeline for hemodynamics simulations: **(A)** Computed Tomography (CT) image; **(B)** Surface mesh after CT segmentation; **(C)** Removal of structures surrounding the left atrium (LA); **(D)** Insertion of cylinders for the pulmonary veins (PV); **(E)** Volumetric LA mesh with two right pulmonary veins (RPVs), two left pulmonary veins (LPVs), a mitral valve (MV) and the left atrial appendage (LAA).

To ensure convergence of hemodynamics simulations, LA meshes from medical image segmentations required several pre-processing phases (see Figure 4). First, a Taubin smoothing filter (with scale factors λ = 0.6, μ = –0.53 and 10 iterations) was applied. Subsequently, cylinders representing the pulmonary veins (PV) and the mitral valve (MV) were manually inserted to the LA mesh using the Autodesk Meshmixer software^2^ so that simulated flow was developed normal to the surface of the vessels. Volumetric meshes were built with a 3D Delaunay refinement algorithm available in the Gmsh software (default parameters) (Geuzaine and Remacle, 2009)^3^ for the subsequent CFD simulations, resulting in the following number of tetrahedral elements for the four LA under study: Patient 1 = 597,550; Patient 2 = 961,015; Patient 3 = 330,091; Patient 4 = 200,951. The whole meshing pipeline was performed with the following Open-Source softwares: Gmsh, Autodesk MeshMixer, MeshLab^4^ and Visual Computing Lab^5^. The reader is referred to García-Isla et al. (2018) for more details of the meshing pipeline.

The studied LAAO devices (see Figure 1) were built using a Computational-Aided Design (CAD) software^6^ and following the on-line product manual and instructions from the companies (Abbott, 2018; Boston Scientific, 2018). Subsequently, several LAAO device configurations, including different size and positioning settings, were exported from the VIDAA platform. Volumetric meshes for all configurations under study were obtained defining two surfaces in Gmsh: one for the LA and another for the implanted device.

#### 2.3.2. Computational Fluid Dynamics

The CFD analysis was performed using ANSYS Fluent 18.2, in which blood was modeled as an incompressible Newtonian fluid (density of ρ= 1,060 kg/m^3^; dynamic viscosity of μ = 0.0035 Pa·s). Blood was simulated using the incompressible Navier-Stokes and continuity equations. Model dynamics was introduced with a sinusoidal function representing a heart cycle of a healthy patient (Fernandez-Perez et al., 2012; García-Isla et al., 2018). Boundary conditions at systole (first 0.40 s) were modeled with the PVs as velocity-inlets and the MV as a wall, to represent that the valve is closed during this cardiac phase (Fernandez-Perez et al., 2012). The opening of the MV in diastole (duration of 0.65 s) was modeled applying an outlet pressure of 8 mmHg (Nagueh et al., 2008) to the MV. All LA walls were modeled as rigid walls with no slip, representing the worst scenario in (persistent) AF, where the atrium barely contracts anymore. The LAAO device was also modeled as a wall to represent that no blood could go through it.

#### 2.3.3. *In silico* Indices for Risk of Thrombus Formation

As in García-Isla et al. (2018), we estimated several *in silico* indices to characterize blood flow patterns derived from CFD simulations and identify pro-thrombotic regions in the LAA, usually where blood flow is complex and velocities are low (Achille et al., 2014). For the sake of simplicity, only the Endothelial Cell Activation Potential (ECAP) index is reported here since it combines the Time-Averaged Wall Shear Stress (TAWSS) with the Oscillatory Shear Index (OSI): low velocity (small TAWSS values) and high complex (large OSI values) blood flows will produce high ECAP values, thus indicating a region with a high risk of thrombus formation.

The equations to derive these indices are included in Appendix 3. For a qualitative analysis, the *in silico* indices were visualized (using ParaView 5.2.0) as colored maps superimposed on the 3D LA geometry, providing an intuitive way to detect regions with irregular blood flow. Furthermore, the distribution in the LAA of the ECAP index for different simulation scenarios was analyzed with histograms showing the percentage of nodes in different ECAP intervals.

#### 2.3.4. Simulation Scenarios

*In silico* blood flow patterns were studied in different simulation scenarios to study the influence of the implanted device configuration. Four simulation scenarios were studied for each patient, resulting in a total number of 16 simulations:

- Without LAAO device (No LAAO).
- LAAO device with size and positioning derived from morphological analysis and criteria defined by experienced clinicians (see section 2.1.1); this is the default configuration shown in the VIDAA platform (VIDAA-init).
- LAAO device with VIDAA-init settings but with a 20% smaller size.
- LAAO device VIDAA-init settings but with a sub-optimal position and orientation (Misplaced).
- LAAO device after manipulation in VIDAA aiming at uniformly covering the union between the LA and LAA (VIDAA-end).

## 3. Results

### 3.1. Blood Flow Velocity Patterns

**Figure 5:**
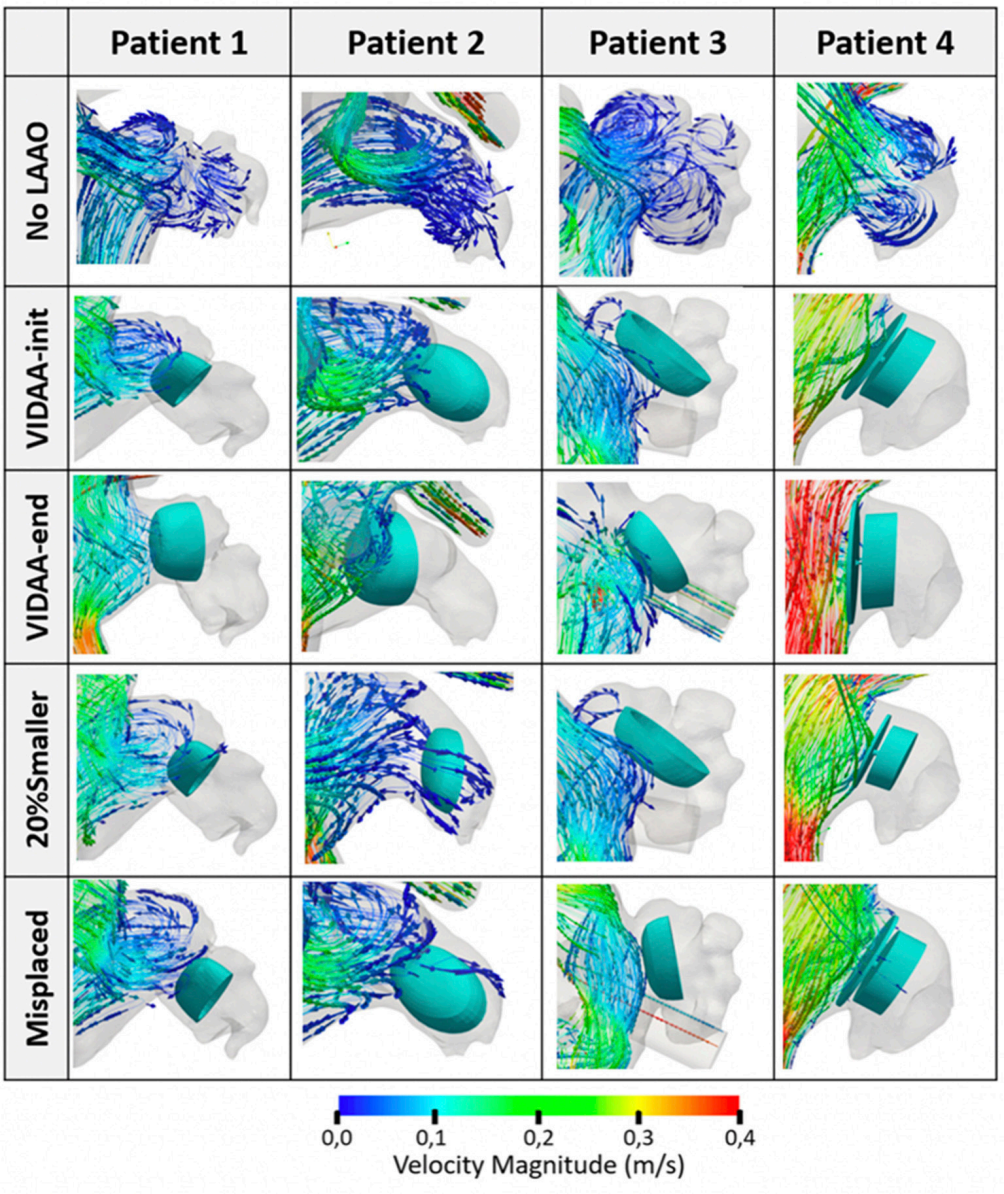
Blood flow streamlines in the left atrial appendage (LAA) from hemodynamics simulations of the four patients under study. Snapshots capture the flow behavior in diastole, when higher velocities are present (*t* = 0.55 s). No LAAO: without device; VIDAA-init: device settings from morphological analysis and clinical recommendations; VIDAA-end: device settings after interactive manipulation in VIDAA; 20% smaller: device with a 20% smaller size than VIDAA-init; Misplaced: device with sub-optimal position and orientation.

Streamlines and velocity vector fields characterizing blood flow patterns in the LA/LAA for different simulation scenarios were analyzed. Figure 5 shows simulation results for the four patients under study and the LAAO configurations described above, in diastole (*t* = 0.55 s, ventricular filling), when blood flow velocities in the LAA are higher. Videos of the hemodynamics simulations (blood flow velocity streamlines for the whole cardiac cycle) for all patients without a device and with the VIDAA-end settings are included as Supplementary Material (see Appendix 2).

At baseline (without an implanted LAAO device, No LAAO configuration, first row in Figure 5), blood flow is entering the LAA in all patients with relatively low velocities, in some cases with complex patterns (e.g., Patient 3) in secondary lobes. The device configuration proposed by the VIDAA platform (both VIDAA-init and VIDAA-end) achieved a perfect occlusion in Patient 1 (second and third row, first column, in Figure 5). On the other hand, blood flow was penetrating the LAA in the same case when decreasing the LAAO device size (20% Smaller scenario), showing a potential stagnation process, with low velocities, in the lateral part of the occluder (blue arrows next to the device; fourth row, first column, in Figure 5). All configurations have the device positioning deeper in the LAA than the VIDAA-end, creating a non-closed cavity between the ostium and the device where new complex blood flow loops appeared. Patient 2 presented a similar behavior to Patient 1 but with a more visible negative effect of reducing the size of the device, allowing more flow through the LAA (fourth row, second column, in Figure 5). On the other hand, Patient 3 (third column in Figure 5) showed a correct flow blockage in all device configurations. Finally, Patient 4 showed fast velocities and laminar flow in the LA cavity outside the implanted device for the VIDAA-end configuration (red streamlines in third row, fourth column in Figure 5), which was not achieved with the remaining settings.

**Figure 6:**
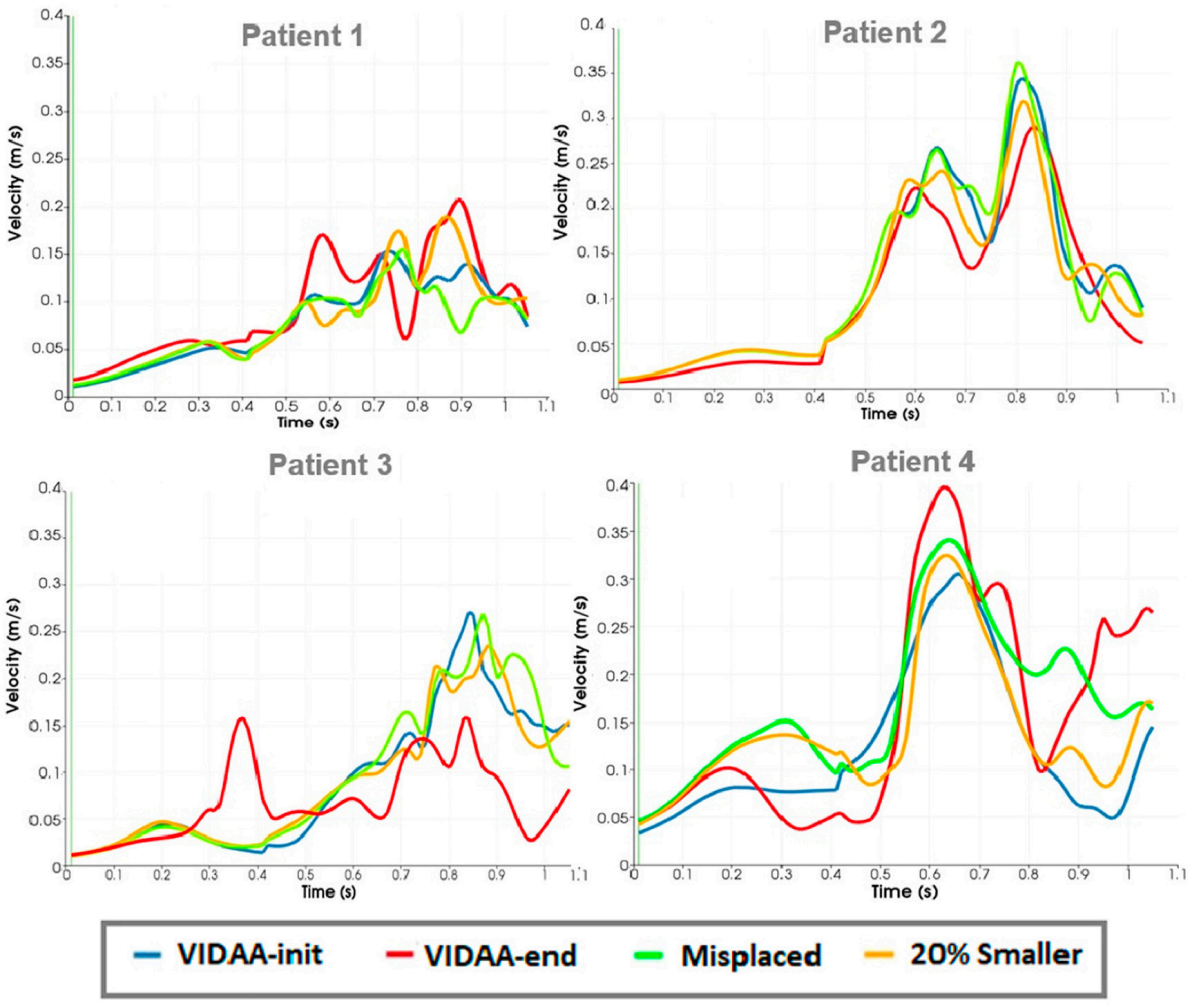
Blood flow velocity curves over the whole cardiac cycle in a point outside the device in the left atrial cavity for all simulation scenarios in the four patients. VIDAA-init: device settings from morphological analysis and clinical recommendations; VIDAA-end: device settings after interactive manipulation in VIDAA; 20% smaller: device with a 20% smaller size than VIDAA-init; Misplaced: device with sub-optimal position and orientation.

Figure 6 depicts blood flow velocity curves over the whole cardiac cycle in a point outside the device (following the LAA centerline direction toward the main LA cavity) for all simulation scenarios in the four patients analyzed in this work. We can appreciate that the VIDAA-end configuration (red curve in Figure 6) leads to velocity profiles with higher magnitudes in diastole for some cases (e.g., see Patient 4), suggesting faster flow circulation and thus less risk of thrombus formation due to device implantation. On the other hand, results are inconclusive for other patients (e.g., see Patient 3), exemplifying the difficulty of characterizing the 4D nature of blood flow patterns by observing 1D velocity profiles in an individual spatial point.

### 3.2. *In silico* Indices for Risk of Thrombus Formation

**Figure 7:**
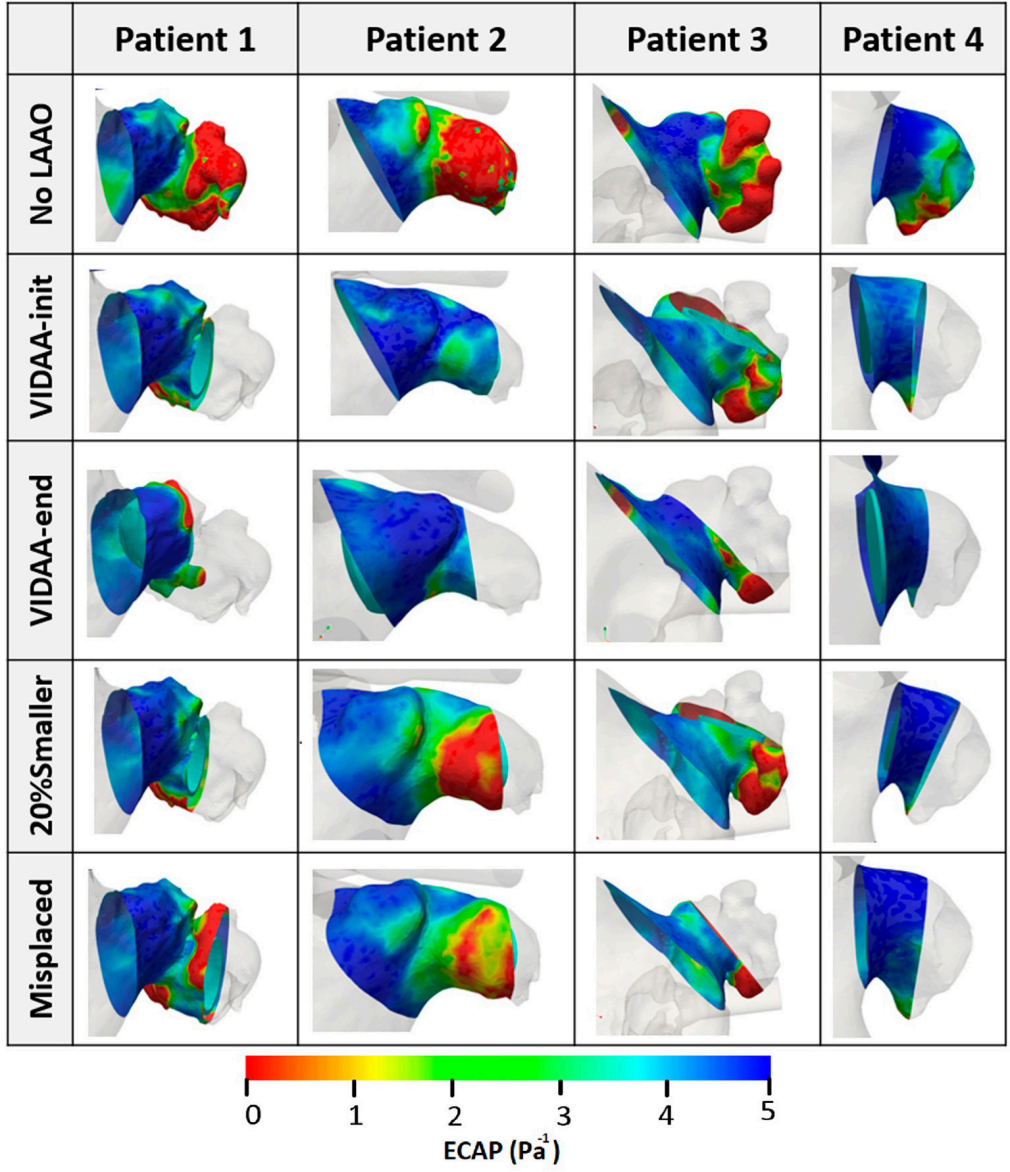
Endothelial Cell Activation Potential (ECAP) distribution (Pa^−1^) visualized as colored maps superimposed to the LA wall for the four patients under study. No LAAO: without device; VIDAA-init: device settings from morphological analysis and clinical recommendations; VIDAA-end: device settings after interactive manipulation in VIDAA; 20% smaller: device with a 20% smaller size than VIDAA-init; Misplaced: device with sub-optimal position and orientation.

Figure 7 shows the ECAP distribution mapped as a colormap onto the LA/LAA geometries for all simulation scenarios in the patients under study. For LAAO scenarios (i.e., all except the No LAAO), the ECAP map is only displayed between the LAA ostium and the device to study the risk of thrombus formation outside the device. It can easily be observed at baseline (No LAAO configuration; first row in Figure 7) that higher values of ECAP (i.e., complex flows and low velocities; red-green areas in Figure 7), thus with a higher risk of thrombus formation, are located in several areas of the LAA depending on morphology complexity. For instance, Patient 4 is the case showing smaller ECAP values due to its smooth morphology and orientation with respect to the main LA cavity, leading to larger blood flow velocity magnitudes in most regions (except in its tip). More importantly, high ECAP areas appear more prominently in device configurations with incorrect LAAO settings (e.g., 20% smaller), whereas the VIDAA-end configuration seems to minimize the presence of these areas.

**Figure 8:**
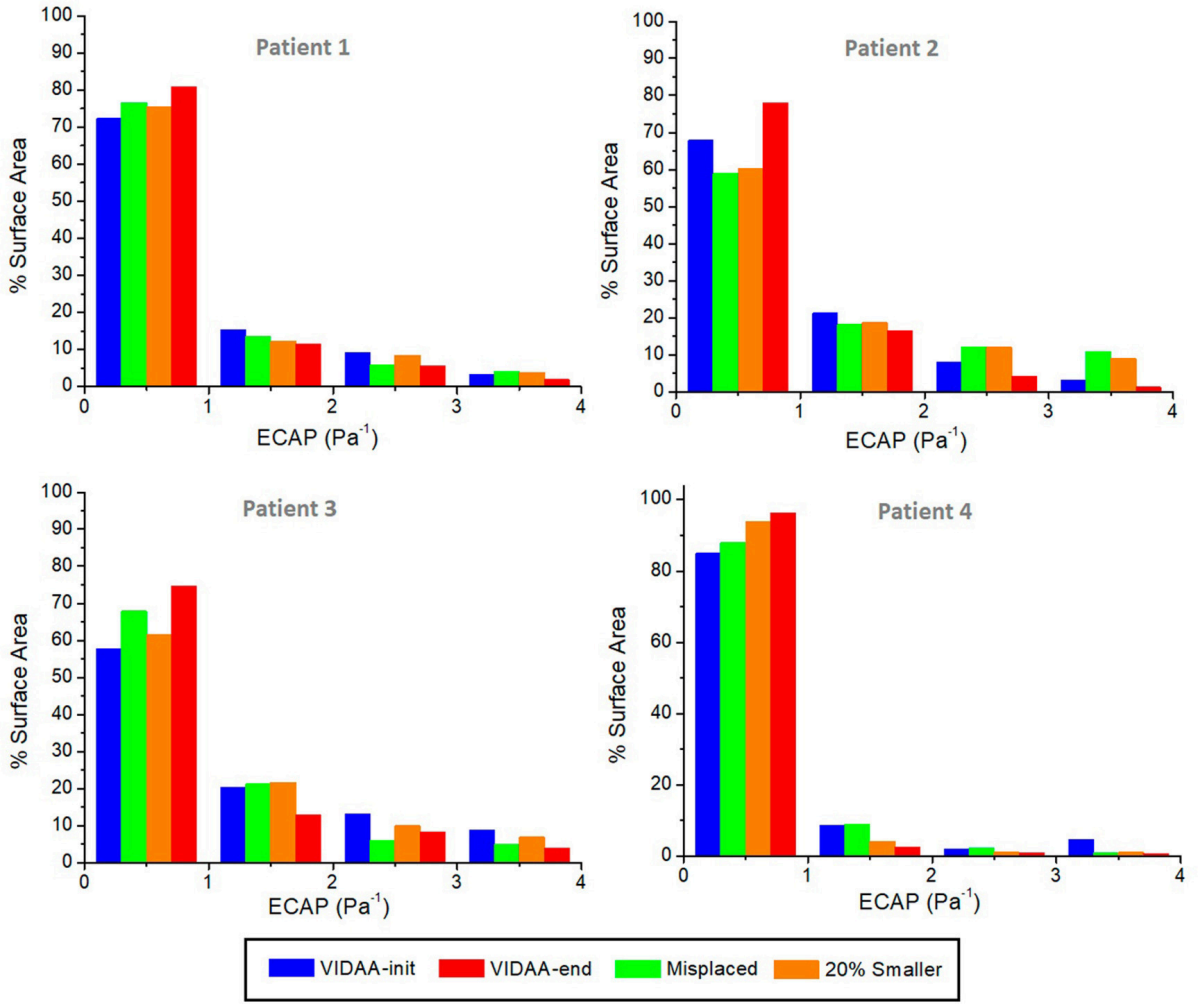
Histograms of Endothelial Cell Activation Potential (ECAP) distribution (Pa^−1^) as a percentage of surface area for each patient and different device scenarios. VIDAA-init: device settings from morphological analysis and clinical recommendations; VIDAA-end: device settings after interactive manipulation in VIDAA; 20% smaller: device with a 20% smaller size than VIDAA-init; Misplaced: device with sub-optimal position and orientation.

Figure 8 plots the histograms of ECAP intervals as percentages with respect to surface area for each patient and different LAAO scenarios. The ECAP maps were analyzed within the region between the device and the LAA ostium. For the four analyzed LAA geometries, the VIDAA-end settings resulted in the lower percentage of surface areas with higher ECAP values (>3). We can also observe that the VIDAA-init settings were not always adequate, sometimes even performing worse than the Misplaced and 20% smaller scenarios, illustrating the benefit of interactively manipulating the device settings in the VIDAA platform.

## 4. Discussion

In this study, different configurations of LAA occlusion devices were evaluated in four patient-specific left atria using advanced computational tools. A web-based platform, so-called VIDAA, was developed in which LAAO devices could be virtually implanted, interactively selecting the desired configuration for a given individual. Once a device configuration was chosen, it was exported out of the platform to built a computational domain where hemodynamics CFD-based simulations were run. The resulting blood flow simulations were post-processed to generate quantitative *in silico* indices (e.g., ECAP) to locally assess the risk of thrombus formation after device implantation, which is not currently possible with existing imaging modalities. Providing such information for different LAAO settings to the clinician would be quite beneficial for a better and faster planning of the intervention.

The implantation of LAAO devices is usually quite successful (around 95%, defined as small flow jets into the LAA after implantation), with peri-procedural complicate rates of approximately 5% (Grosset-Janin et al., 2015). Nevertheless, residual bleedings are not uncommon and the reduction of thrombus formation risk, even if superior to OAC-based therapy, could be improved (60–75% Grosset-Janin et al., 2015). Moreover, it is not well-known when patients can stop anti-coagulants after LAAO implantation, which can lead to device-related thrombus formation (7% of cases in a recent study Fauchier et al., 2018). The development of advanced computational tools including 3D medical imaging, clinician-friendly interfaces and biophysical modeling to fully characterize the LAA 3D morphology, 4D blood flow patterns and virtually predict the individual risk of thrombus formation for different device settings, would contribute to the acceptance of LAAO therapies, properly assessing their cost-effectiveness and clinical benefit as well identifying their limitations, which is still under debate (Hu and Yogeswaran, 2015; Mandrola et al., 2018).

The obtained results confirm the relevance of an appropriate LAAO device configuration, personalized to each patient, to ensure a complete occlusion of the LAA cavity and minimize the creation of areas around the implanted device prone to thrombus formation (e.g., low velocities and complex flow). Consistently, LAAO settings obtained after device manipulation in the VIDAA platform (VIDAA-end) were the most appropriate, ensuring a complete blockage of the LAA cavity in all cases. On the contrary, other LAAO configurations, including the VIDAA initial configuration based on LAA morphological analysis and clinical recommendations (VIDAA-init), repeatedly created complex flow loops with low velocities around the device, incrementing the risk of thrombus formation post-intervention. However, the VIDAA-end positioning of the device may not be adequate if it leads to a blockage of the circumflex artery, which is located just outside the LAA ostium. Unfortunately, we did not have information about the circumflex artery exact location in the studied cases. In the future, this information will be obtained from CT images and added as an additional constraint in the VIDAA platform.

Device sizing and positioning are then crucial, assuming a good attachment to the LAA wall. Nowadays, the process for selecting the LAAO size and defining the landing zone where the device will be released is too subjective and prone to errors due to the lack of quantitative techniques and high-resolution 3D images. We have observed in our study that small changes in the selection of transversal 2D planes along the LAA centerline produced maximum diameters in the range of 22–31 mm in the same geometry. Similar variability can be found when using 2D-based or different medical imaging modalities, as it was demonstrated in López-Mínguez et al. (2014), where LAAO dimensions were consistent from X-ray, echocardiography and CT images in only 23% of the cases. Therefore, a full 3D characterization of the LA/LAA geometry (i.e., dimensions, relative orientation) is required for an optimal device sizing/positioning and avoid generating areas between the implanted device and the atrial wall prone to thrombus formation. A reduced learning curve of the LAAO device implantation process and more comprehensive intervention planning has already been demonstrated by using CT images (Wang et al., 2016; Chow et al., 2017). Recently (De Potter et al., 2018), 3DRXA has also been proven an interesting alternative to echocardiographic images during the intervention.

The VIDAA platform has then been developed to allow the interactive 3D visualization of LA/LAA geometries and CAD models of the devices. The integration of VIDAA with hemodynamics simulations has been very helpful to identify areas with high risk of thrombus formation under certain LAAO configurations. The ECAP parameter, initially proposed by Achille et al. (2014) in an aortic aneurysm application, provided consistent results, with higher values in the LAA and showing its potential to estimate thrombogenesis. Overall, the VIDAA platform and biophysical models should provide a more complete way to analyze the relation between LAA shape, hemodynamics, device settings and thrombus formation to the clinicians prior to the intervention.

The presented methodology has several limitations that could be improved in the future. For instance, the 3D model construction, including image processing steps (e.g., segmentation, definition of ostium plane) and the meshing pipeline, is semi-automatic, which could lead to intra-operator inconsistencies. The obtained image processing results and corresponding 3D models were qualitatively evaluated by several observers for each case. However, even if developing a fully automatic image processing pipeline was out of the scope of this paper, left atrial segmentation techniques based on deep-learning algorithms are already becoming available (see recent Atrial Challenge at STACOM18 workshop^7^). Sensitivity analysis and convergence studies will also identify the most critical meshing pipeline parameters, such as the required mesh resolution, to obtain robust wall shear stress maps, which is critical to estimate reliable ECAP maps.

Moreover, several boundary conditions and modeling choices could be upgraded to achieve more realistic simulations. The assumption of rigid LA walls for a persistent AF patient is valid from a clinical point of view. Nevertheless, the LAA is quite a flexible structure and the mitral ring displacement is non-negligible to properly simulate LA reservoir, conduit and booster pump phases. Motion-based constraints could be imposed from patient-specific information, as in Otani et al. (2016) from CT images or similar. Moreover, boundary conditions such as the inlet velocity profile should be adapted to atrial fibrillation (i.e., absence of atrial contraction). More sophisticated numerical techniques such as Fluid-Structure interaction (between blood flow and LA wall), contact-based (between device and LA wall) or discrete particle models (for coagulation and thrombus formation Hathcock, 2006) may provide more realistic simulations, at the expense of increased computational costs and complexity. In addition, patient-specific blood flow boundary conditions (e.g., based on Doppler data), rather than the generic curves used in this study, would be required for an individualized assessment of hemodynamics.

The post-processing of CFD simulations has been one of the most critical steps in the developed modeling pipeline. For instance, the visualization of blood flow streamlines is highly dependent on the location of the seeds required to generate them. An incorrect seed placement can lead to misinterpretation of CFD simulations and wrong conclusions. To study LAA blood flow patterns in the LAA it is convenient to place seeds near the LAA ostium, according to our experience. Additionally, one needs to be cautious on using velocity 1D profiles and values evaluated in a single point in space as an estimation of blood flow pattern behavior. It is not obvious to select a point in which reliably comparing velocity profiles for different device configurations, as shown in Figure 6. This is also critical for the validation of simulation results with clinical data. We have performed initial visual comparisons between mitral valve and LAA velocity profiles from our simulations and from Doppler data of the studied patients, showing promising similarities. Nevertheless, a more exhaustive and quantitative validation with more complete Doppler or 4D-flow MRI data, ideally on several geometries and with different LAAO devices, is still missing. In addition, the analysis of a larger number of cases would allow identifying patients with thromboembolic history and compare their morphological and hemodynamics parameters given by VIDAA and biophysical models with patients without a thrombus.

## 5. Conclusions

The VIDAA platform was developed to perform virtual implantation of LAAO devices on patient-specific geometries in an interactive way. Its combination with biophysical models of hemodynamics and complete LAA shape 3D characterization offers clinicians unprecedented computational tools to select the optimal device settings prior to the intervention. Different LAAO device settings can be tested to minimize the areas prone to thrombus formation after device implantation, according to *in silico* indices derived from CFD simulation results. The LAAO configurations found after manipulation of device settings in the VIDAA platform were linked to a reduced risk of thrombus formation outside the implanted device, according to a qualitatively analysis of blood flow streamlines and ECAP maps. Future work will be focused on the evaluation of the VIDAA platform in a clinical environment, analyzing a larger number of LAA cases and exploring its use as an alternative to 3D printing for interventional planning, also in combination with immersive visualization interfaces.

## Data Availability

Simulation data including solver configurations and results are available under request. A beta version of the VIDAA platform is available at the GitHub account of the BCN-Medtech research unit (https://github.com/bcnmedtech).

## Author Contributions

AA generated CFD models, processed and analyzed data, developed VIDAA platform, and wrote the paper. AO conceived the research and VIDAA platform, designed CFD models and analyzed data, wrote the paper. CY developed VIDAA platform, gave technical support on the web tools. ES provided clinical data from OLV and performed segmentation of 3DRA images. MN-G gave technical support on LA and LAA mesh processing. AF-Q computed LAA morphological measures and created database. JM gave technical support on reconstruction of CT images and CFD models. IG developed the algorithm for centreline extraction. TDP provided data from OLV and practical context. DA provided data from Hospital de la Santa Creu i Sant Pau of Barcelona and set specifications for VIDAA platform. XF provided data from Hospital Clínic de Barcelona and set specifications for VIDAA platform. OC oversaw the whole project, conceived the research, and wrote-revised the paper.

### Conflict of Interest Statement

The authors declare that the research was conducted in the absence of any commercial or financial relationships that could be construed as a potential conflict of interest.

## Footnotes

^1^www.itksnap.org

^2^http://www.meshmixer.com/

^3^http://gmsh.info/

^4^http://www.meshlab.net/

^5^http://vcg.isti.cnr.it/

^6^Geometry-Design Modeler (Ansys 18.2)

^7^http://atriaseg2018.cardiacatlas.org/
