# Supplementary material for: In silico Optimization of Left Atrial Appendage Occluder Implantation Using Interactive and Modeling Tools
Source: Front Physiol. 2019 Mar 22;10:237. doi: 10.3389/fphys.2019.00237 (PMC6440369; doi:10.3389/fphys.2019.00237)
Supplement: Supplementary file 1 [file Presentation_1.pdf]

Supplementary Material:

1 SUPPLEMENTARY DATA

APPENDIX SECTION

Appendix 1: Device Selection

Table A.1. This table shows the optimal left atrial appendage occlusion (LAAO) device sizes based on LAA diameters at the landing zone (LZ). Maximum and mean diameters at the LZ are used to select the Watchman and the Amplatzer Amulet devices, respectively.

Watchman		Amplatzer Amulet		
LZ range (mm)	Device size (mm)	LZ range (mm)	Device size (mm)	Disc Diameter (mm)
16.8 - 19.3	21	11-13	16	22
19.2 - 22.1	24	13-15	18	24
21.6 - 24.8	27	15-17	20	26
24.0 - 27.6	30	17-19	22	28
26.4 - 30.4	33	19-22	25	32
26.4 - 30.4	33	22-25	28	35
-	-	25-28	31	38
-	-	28-31	34	41

Appendix 2: Audiovisual material

- Demonstration of VIDAA platform (*Video 1.mp4*).
- Hemodynamics simulations (blood flow velocity streamlines) for Patient 1:
  - Without device (*Video 2.mp4*).
  - VIDAA-end device configuration (*Video 3.mp4*).
- Hemodynamics simulations (blood flow velocity streamlines) for Patient 2:
  - Without device (*Video 4.mp4*).
  - VIDAA-end device configuration (*Video 5.mp4*).
- Hemodynamics simulations (blood flow velocity streamlines) for Patient 3:
  - Without device (*Video 6.mp4*).
  - VIDAA-end device configuration (*Video 7.mp4*).
- Hemodynamics simulations (blood flow velocity streamlines) for Patient 4:
  - Without device (*Video 8.mp4*).
  - VIDAA-end device configuration (*Video 9.mp4*).

Appendix 3: Definition of in silico hemodynamics indices.

The time-averaged wall shear stress magnitude (TAWSS) was defined as:

$$TAWSS = \frac{1}{T}\int_{0}^{T} |WSS| dt, \tag{S1}$$

where T was the period of the cardiac cycle. The TAWSS captures the mechanobiological effects of the WSS in the LAA wall: small values of WSS represent low flow velocities, which are related with blood stasis and higher coagulation risk. The oscillatory shear index is a non-dimensional parameter characterizing the predominant direction of the blood during the cardiac cycle and was defined as:

$$OSI = \frac{1}{2}\frac{\int_{0}^{T} WSSdt}{\int_{0}^{T} |WSS|dt}. \tag{S2}$$

In other words, the OSI parameter estimates the WSS field direction changes due to complex flow pattern, having a maximum value of 0.5. The endothelial cell activation potential is the ratio between the OSI and the TAWSS:

$$ECAP = \frac{OSI}{TAWSS}, \tag{S3}$$

which could identify regions in the left atria with high risk of thrombus formation (high ECAP values are obtained from high OSI or complex flows and low TAWSS or low blood flow velocities).
